# Response network analysis of differential gene expression in human epithelial lung cells during avian influenza infections

**DOI:** 10.1186/1471-2105-11-170

**Published:** 2010-04-06

**Authors:** Ken Tatebe, Ahmet Zeytun, Ruy M Ribeiro, Robert Hoffmann, Kevin S Harrod, Christian V Forst

**Affiliations:** 1 Department of Clinical Sciences, University of Texas Southwestern Medical Center, Dallas, TX, USA; 2 Bioscience Division, Los Alamos National Laboratory, Los Alamos, NM, USA; 3 Theoretical Biology and Biophysics, Los Alamos National Laboratory, Los Alamos, NM, USA; 4 Computational Biology Center, Memorial Sloan Kettering Cancer Center, New York, NY, USA; 5 Lovelace Respiratory Research Institute, Albuquerque, NM, USA

## Abstract

**Background:**

The recent emergence of the H5N1 influenza virus from avian reservoirs has raised concern about future influenza strains of high virulence emerging that could easily infect humans. We analyzed differential gene expression of lung epithelial cells to compare the response to H5N1 infection with a more benign infection with Respiratory Syncytial Virus (RSV). These gene expression data are then used as seeds to find important nodes by using a novel combination of the Gene Ontology database and the Human Network of gene interactions. Additional analysis of the data is conducted by training support vector machines (SVM) with the data and examining the orientations of the optimal hyperplanes generated.

**Results:**

Analysis of gene clustering in the Gene Ontology shows no significant clustering of genes unique to H5N1 response at 8 hours post infection. At 24 hours post infection, however, a number of significant gene clusters are found for nodes representing "immune response" and "response to virus" terms. There were no significant clusters of genes in the Gene Ontology for the control (Mock) or RSV experiments that were unique relative to the H5N1 response. The genes found to be most important in distinguishing H5N1 infected cells from the controls using SVM showed a large degree of overlap with the list of significantly regulated genes. However, though none of these genes were members of the GO clusters found to be significant.

**Conclusions:**

Characteristics of H5N1 infection compared to RSV infection show several immune response factors that are specific for each of these infections. These include faster timescales within the cell as well as a more focused activation of immunity factors. Many of the genes that are found to be significantly expressed in H5N1 response relative to the control experiments are not found to cluster significantly in the Gene Ontology. These genes are, however, often closely linked to the clustered genes through the Human Network. This may suggest the need for more diverse annotations of these genes and verification of their action in immune response.

## Background

Techniques such as microarray analysis allow measurements of the differential gene expression in cells for tens of thousands of genes simultaneously. The ability to measure changes in the transcription activity of a cell in response to an external stimulus allows for a system-wide approach in which pathways and sub-networks are analyzed rather than the activity of isolated genes [1]. Further, the development of biological knowledge systems such as the Gene Ontology (GO) [2] have provided a framework in which groups of genes can be classified in three areas: biological processes, molecular function and cellular components. This ontological classification scheme of gene function gives a hierarchical context in which groups of genes can be regarded to determine how closely they are functionally related [3].

A complimentary approach to above classification based on GO is the assessment of molecular functions in the context of known interactions between genes, DNA/RNAs, proteins and small chemicals, as mapped in biochemical interaction maps, pathways and networks [4]. The novel combination of these biochemical networks, along with the classifications provided by the GO, allows important clusters of genes in the cellular response to be identified. It further provides evidence by adjacency and pathway connectivity to assign genes that may not be significantly expressed to the relevant gene clusters.

Here, these methods are used to study properties of avian influenza infection with H5N1 virus, and to compare this infection to another upper respiratory tract infection, namely respiratory syncytial virus (RSV). The H5N1 influenza virus shows remarkably higher virulence than other strains of influenza [5]. A greater understanding of the H5N1 virus is motivated by epidemiological concerns, particularly in-light of the recent emergence of the H1N1 strain, because it is a prime candidate as a future pandemic infection should it mutate or reassort into a form that can be easily contracted by humans [6,7].

As a cross check on the significance analysis, a support vector machine (SVM) algorithm [8] is used to identify which genes show the greatest differences in their expressions between the H5N1 infected and control samples. This ranking is then compared to the significance analysis and genes associated with over represented GO nodes as well as genes that are closely associated with significant genes through interactions in the Human Network. This cross check will allow us to identify nodes that are not properly annotated, thus are not captured by the GO analysis.

## Results and Discussion

Results are presented in threefold. First, significant clustering of genes into sub-graphs of the Gene Ontology are identified using the GO enrichment analysis plugin *BiNGO *[9] for *CytoScape *[10]. Second, many of the most strongly up and down regulated genes are examined and their possible function in the cellular response of Normal Human Bronchial Epithelial (NHBE) cells to H5N1 is considered with comparison to RSV and Mock infections. Comparisons were made at 8 and 24 hours postinfection for comparison of gene expression changes prior to and following productive viral replication, generally 12-14 hours postinfection in this NHBE culture model. Viral replication was verified by standard plaque assay techniques under the appropriate biocontainment levels. A total of 138 and 213 genes were found to be both significant biologically and statistically post infection at 8 and 24 hours, respectively. Of these, only a small fraction are associated with over represented GO nodes. Third, human response networks are calculated and correlated with GO.

### Significant Sub-Graphs

**Figure 1:**
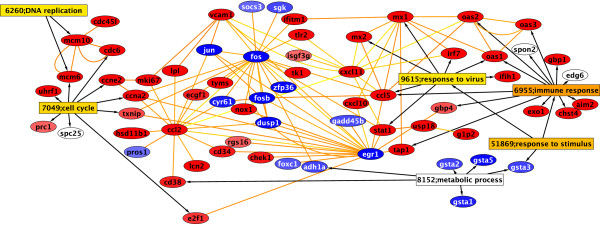
**Network of significant genes and GO nodes at 24 hours**. The Gene Ontology nodes found to be significant in the BiNGO analysis are shown as rectangles, with the more orange nodes being more statistically significant. The genes associated with the GO nodes are listed in ovals connected by black arrows to the GO nodes. These genes are further connected to other genes in the Human Network via yellow and orange edges. Red ovals indicate up-regulated genes while blue indicates down-regulated genes (see Additional files 1 &2).

After 8 and 24 hours of H5N1 exposure, 138 and 213 significant genes relative to the controls were identified, respectively. In spite of 138 genes being found at 8 hours, no significant over-representation of any GO nodes was found by BiNGO. At 24 hours after infection, several GO nodes showed over-representation and are listed in Table 1 along with the significant genes that are annotated with the corresponding GO ID. Only nodes that are significant at the 0.05 level using a Bonferroni correction are listed. Using the more liberal Benjamini correction did not yield appreciably different graphs [11]. The genes associated with each of these GO nodes and their functional interactions with other genes as described by our constructed Human Network are shown in Figure 1 (see Additional Files 1 and 2). Most of the genes in the GO clustering and those found associated/related in the Human Network are up-regulated, with a minority being down-regulated. Many of the genes with the greatest change in expression, however, were down regulated such as EGR2, FOS and EGR1 as shown in Table 2 (see Table 3 for a list of gene-definitions).

**Table 1:** Significant GO nodes

Significant GO Nodes at 24 Hours		
**GO ID**	**GO description**	***p*-value**

**gene**	**gene description**	**log**_2_**(avg. fold change)**

**6955**	**Immune response**	*p *= 6.23 × 10^-16^
AIM2	absent in melanoma 2	2.71
CCL5	chemokine (C-C motif) ligand 5	1.64
CHST4	carbohydrate (N-acetylglucosamine 6-O) sulfotransferase 4	1.53
EDG6	endothelial differentiation, lysophosphatidic acid GPCR 6	-1.22
EXO1	exonuclease 1	0.08
GBP1	guanylate binding protein 1, interferon-inducible, 67 kDa	1.75
GBP4	guanylate binding protein 4	1.25
OAS1	2',5'-oligoadenylate synthetase 1, 40/46 kDa	1.91
OAS2	2',5'-oligoadenylate synthetase 2, 69/71 kDa	2.36
OAS3	2',5'-oligoadenylate synthetase 3, 100 kDa	2.68
SPON2	spondin 2, extracellular matrix protein	-1.29
TAP1	transporter 1, ATP-binding cassette, sub-family B (MDR/TAP)	1.63
**7049**	**Cell Cycle**	*p *= 1.88 × 10^-10^
CCNA2	cyclin A2	1.58
CCNE2	cyclin E2	2.32
CDC6	CDC6 cell division cycle 6 homolog (*S. cerevisiae*)	1.86
E2F1	E2F transcriptoin factor 1	1.37
MCM6	MCM6 minichromosome maintenance deficient 6	2.05
PRC1	protein regulator of cytokinesis 1	1.17
SPC25	kinetochore protein	2.15
TXNIP	thioredoxin interacting protein	1.29
UHRF1	ubiquitin-like, containing PHD and RING finger domains, 1	1.63
**9615**	**Response to Virus**	*p *= 5.98 × 10^-9^
CCL5	chemokine (C-C motif) ligand 5	1.64
IFIH1	interferon induced with helicase C domain 1	1.87
IRF7	interferon regulatory factor 7	1.76
MX1	myxovirus (influenza virus) resistance 1, interferon-inducible prot. p78 (mouse)	2.57
MX2	myxovirus (influenza virus) resistance 2 (mouse)	2.43
OAS1	2',5'-oligoadenylate synthetase 1, 40/46 kDa	1.91
STAT1	signal transducer and activator of transcription 1, 91 kDa	1.66
**6260**	**DNA Replication**	*p *= 8.92 × 10^-9^
MCM6	MCM6 minichromosome maintenance deficient 6	2.05
MCM10	MCM10 minichromosome maintenance deficient 10 (*S. cerevisiae*)	2.10

The most significant GO nodes are given with their raw p-values. Under each GO entry is a list of the genes associated with that node.

**Table 2:** Top 3 most strongly regulated genes at 8 and 24 hours

Gene	8 hrs	24 hrs
EGR2	-3.65 ± 0.10	-3.47 ± 0.00
FOS	-3.47 ± 0.00	-3.25 ± 0.16
EGR1	-3.32 ± 0.00	-3.10 ± 0.08

Errors are based on the variation of the fold change in expression between the three independent trials. EGR2 has the greatest average down-regulation of all genes observed in this study at both 8 and 24 hours with FOS being more down-regulated than EGR1 at 8 hours and FOS and EGR1 switching places at 24 hours. The log_2 _fold changes are shown.

**Table 3:** Definition of genes referred to in the main text

Gene	Definition
ABCC3	ATP-binding cassette, sub-family C (CFTR/MRP), member 3
CCL2	chemokine (C-C motif) ligand 2
CDC6	cell division cycle 6 homolog (*S. cerevisiae*)
CTGF	connective tissue growth factor
CXCL11	chemokine (C-X-C motif) ligand 11
CYR61	cysteine-rich, angiogenic inducer, 61
DUSP1	dual specificity phosphatase 1
EGR1/2	early growth response 1/2
FOS	v-fos FBJ murine osteosarcoma viral oncogene homolog
FOSB	FBJ murien osteosarcoma viral oncogene homolog B
IFI27	interferon, alpha-inducible protein 27
IFIT5	interferon-induced protein with tetratricopeptide repeats 5
IL17C	interleukin 17C
ISG15	ISG15 ubiquitin-like modifier
JUN	v-jun sarcoma virus 17 oncogene homolog (*avian*)
LGALS2	lectin, galactoside-binding, soluble, 2
MCM6	minichromosome maintenance deficient 6
PLK3	polo-like kinase 3 (*Drosophila*)
PTGS2	prostaglandin-endoperoxide synthase 2
SDPR	serum deprivation response (phosphatidylserine binding protein)
SOCS1/3	suppressor of cytokine signaling 1/3
STAT1	signal transducer and activator of transcription 1, 91 kDa
STAT3	signal transducer and activator of transcription 3 (acute-phase response factor)
TLR	toll-like receptor
TAP1	transporter 1, ATP-binding cassette, sub-family B (MDR/TAP)
ZFP36	zinc finger protein 36, C3H type, homolog (*mouse*)

**Figure 2:**
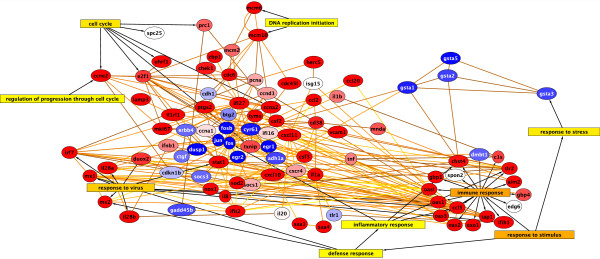
**Network of genes using daughters of BiNGO nodes as seeds**. Genes that are up-regulated are shown in red and those in blue are down-regulated. Any gene found to be a daughter of a GO node found to be significant under the BiNGO analysis was included in a seed set used to generate the resulting network connecting the BiNGO nodes. In this case, the seed genes are not necessarily significant, rather, it is their associated GO node that is significant (see Additional files 3 &4).

Another visualization of these results is depicted in Figure 2 (see Additional files 3 &4), which contains a network that used daughters of BiNGO nodes as seeds for the human response network. As a result there are many genes connected to each GO node. The increased number of paths between GO nodes obscures the genes that are at the hub of activity, though FOS, IFI27, STAT1, CXCL11 and CDC6 are rather well connected. This is in contrast to Figure 1, which shows a graph where the significant genes were used as seeds regardless of their GO connections to generate a human response network, and the resulting graph was then merged with the GO nodes found to be significant with BiNGO.

Interestingly, no significant over-representation of GO nodes can be found among the significantly represented genes during RSV infection. That is, looking at the genes that are both biologically and statistically significant in the RSV but not in the Mock assays. This may indicate that the response to RSV at times earlier than 24 hours does not involve activating genes that are not part of the normal metabolic behavior of the cell. It is possible that there is a more subtle interplay between the gene activations in how the cell orchestrates a response. Alternatively, the effects of early RSV infection could be small such that the cell has not mustered significant changes in gene expression by 24 hours post infection. In any case, using the union of all genes found to be significant in any of the control experiments is rather conservative and may exclude sufficient true positives to make finding significantly over represented GO nodes difficult; however, this also highlights the dramatic response of the cell to infection by H5N1.

### Genes of Interest

Although no significant GO nodes are found at 8 hours, there is one sub-network associated with binding in the Gene Ontology that contains a fair number of genes. At 8 hours this sub-network contains a handful of genes including CTGF, CYR61, SDPR and LGALS2. By 24 hours this has expanded to accommodate numerous genes. This parent node common to the genes of this subnetwork is associated with IFIT5, a gene that codes for an interferon induced protein. Other subnetwork that are easily identified by visual inspection include genes involving transcription regulation, with the genes EGR1, JUN, MCM6, FOSB, FOS and ZFP36 being significantly regulated at both 8 and 24 hours, and genes involved with post transcriptional/translational modification such as TAP1, ISG15, and PLK3. There are also a number of kinase/phosphatase related genes, DUSP1, ABCC3, SOCS3. Among these, several of these genes also appear among the top 35 components to the vector normal to the OHP in the SVM analysis. Table 4 lists genes from Table 5 that appear as significant in the 8 and 24 hour experiments. Although most of these genes are not found in the BiNGO analysis, they are generally found to be highly connected to those genes through the Human Network. Many of these genes, including CYR61, EGR1, JUN and DUSP1 are prominently displayed in the response network in Figure 1.

**Table 4:** Genes common to significance and SVM analyses

SVM Gene	**log**_2_**(Fold Change)**	Description (if available)	8 hrs	24 hrs
CYR61	-1.51	cysteine-rich, angiogenic inducer, 61	+	+
SOCS3	-1.06	suppressor of cytokine signaling 3	-	+
AK024238	-1.32	mRNA, possibly cadherin 6, type 2 (CDH6)	+	+
AA768672	-1.47	mRNA, possibly LSM14A	+	+
THC2348879	-1.22		+	-
THC2313287	-2.74		+	+
IER2	-1.64	immediate early response 2	+	+
ZFP36	-1.74	zinc finger protein 36	+	+
FOS	-3.47	v-fos FBJ murine osteosarcoma viral oncogene homolog	+	+
NFKBIE	1.08	nuclear factor of kapppa light polypeptide gene enhancer in B-cells inhibitor	+	+
AT_nC_3	-1.32		+	-
CTGF	-1.43	connective tissue growth factor	+	-
AF159295	-1.89	mRNA, MAP/microtubule affinity-regulating kinase 3 (MARK3)	+	+
JUN	-1.74	v-jun sarcoma virus 17 oncogene homolog (*avian*)	+	+
FOSB	-2.18	FBJ murien osteosarcoma viral oncogene homolog B	+	+
EGR1	-3.18	early growth response 1	+	+
HP1BP3	-1.00	heterochromatin protein 1, binding protein 3	+	-
DUSP1	-1.79	dual specificity phosphatase 1	+	+
ABCC3	1.38	ATP-binding cassette, sub-family C (CFTR/MRP), member 3	+	+
FOXC1	-1.15	forkhead box C1	-	+
SFRS5	-1.47	splicing factor, arginine/serine-rich 5	+	+
DB363693	-1.43		+	-
THC2343678	-1.47		+	+
A_24_P161068	-1.09		-	+

Genes are listed in order of importance in the SVM analysis. The fold change in each gene is the average fold change in gene expression including both the 8 and 24 hour data. In the last two columns, a "+" indicates that the gene was found in both the Significance and SVM Analyses at 8 or 24 hours, respectively. A "-" indicates that the gene was not found in the results in both analyses at 8 or 24 hours. Genes through sfrs5 are among the top 35 and through the end of the list are in the top 43.

**Table 5:** Components of the Optimal Hyper Plane (OHP) in the SVM analysis

SVM Components of Significant Genes					
**Gene**	**log**_2_**(Exp Fold)**	**Ctrl Fold**	**Exp pval**	**Ctrl pval**	**Component**

CYR61	-1.51	0.16	2.8 × 10^-6^	0.31	-.0098
SOCS3	-1.06	-0.07	0.0017	0.39	-0.0095
LOC196752	-0.94	0.11	0.01	0.6	-0.0094
ALDH3B1	-0.86	0.07	0.012	0.9	-0.0094
KLF6	-1.60	0.26	5 × 10^-7^	0.38	-0.0093
AK024238	-1.32	0.06	7.6 × 10^-7^	0.39	-0.0093
AA768672	-1.47	-0.30	6.9 × 10^-7^	0.3	-0.0093
TUBAL3	-1.22	-0.62	0	0.078	-0.0092
THC2348879	-1.22	-0.09	1.4 × 10^-6^	0.41	-0.0088
THC2313287	-2.74	-0.09	4.1 × 10^-13^	0.52	-0.0088
IER2	-1.64	0.07	1.8 × 10^-6^	0.61	-0.0088
ZFP36	-1.74	0.10	3.7 × 10^-7^	0.67	-0.0086
FOS	-3.47	0.10	1.4 × 10^-19^	0.6	-0.0086
AHNAK	-0.94	0.15	0.012	0	-0.0085
NFKBIE	1.08	-0.09	0	0.37	0.0085
A_24_P7785	-0.97	-0.12	0.013	0.4	-0.0085
AT_nC_3	-1.32	-0.20	0.0067	0.78	-0.0084
CTGF	-1.43	0.03	2 × 10^-8^	0.37	-0.0084
AT_ssH_RR_5	-1.56	-0.14	1.3 × 10^-6^	0.26	-0.0083
AF159295	-1.89	0.26	2.5 × 10^-7^	0.3	-0.0083
JUN	-1.74	-0.20	7.3 × 10^-7^	0.26	-0.0083
FOSB	-2.18	0.26	1.2 × 10^-9^	0.58	-0.0082
SYT12	0.88	-0.09	0.012	0.93	0.0082
AK022038	-0.89	0.26	0.013	0.35	-0.0082
EGR1	-3.18	0.21	1.9 × 10^-18^	0.44	0.0081
A_24_P494658	-0.86	0.08	0.022	0.25	-0.008
HP1BP3	-1.00	-0.04	0.0033	0.62	-0.008
ENST00000380874	-0.97	-0.42	0.0033	0.21	-0.0079
TRAF2	-0.77	-0.17	0.025	0.31	0.0078
DUSP1	-1.79	0.43	2.2 × 10^-7^	0.27	-0.0078
RELB	1.14	0.15	0	0.29	0.0078
ABCC3	1.38	0.03	1.9 × 10^-6^	0.23	0.0078
FOXC1	-1.15	0.01	0	0.72	-0.0077
SFRS5	-1.47	0.41	7.1 × 10^-9^	0.31	-0.0077
G0S2	2.38	0.42	1.5 × 10^-8^	0.22	0.0076

The components of the OHP in the SVM analysis are displayed along with the average fold change of the gene in the H5N1 data and control data, and the average p-values in the H5N1 and control data. The length of each component is also given. A negative component to the vector normal to the OHP indicates a decrease in the fold change in gene expression from the control to the H5N1 exposures while a positive component indicates an increase in the differential expression from control to H5N1.

Among the three most dramatically regulated genes at both 8 and 24 hours is the Early Growth Response 1 gene EGR1, which is down-regulated by about an order of magnitude at both 8 and 24 hours. Other studies of epithelial lung cells in mice have shown significant up regulation of this gene in association with lung injury, though on much shorter time scales [12]. Up-regulation of EGR1 and EGR2 have been seen after wounding in mouse studies at several stages of development from embryo to adult, though knockout studies show that neither gene is essential to healing [13]. This suggests a redundancy in systems in the cellular response to trauma. Such backup systems are excluded in this study by the selection criteria as a gene must appear in all three experimental sets to be selected. The timescale for EGR1 up-regulation in these cases is much faster and is reported to be insignificant 90 min post trauma. Similarly, EGR2 was reported to decrease to near zero after 1 hour. EGR1 is a master transcription factor that has been shown to be up-regulated by histamine in human aortic endothelial cells [14]. It is not known whether this effect also exists in pulmonary endothelial cells, but based on these data this seems to be a possibility. It is curious, however, that this study finds EGR1 to be strongly down regulated, in contrast to other studies. The immediate early gene EGR1 is also activated by injury, suggesting that mechanical stress of the cellular membrane and viral infection may have a common factor in the response triggered in the cell [15]. There are also significant differences between an intense, onetime injury and the more gradual onset and constant stress of a viral infection. These differences may help explain the faster timescales of gene activation associated with injury.

**Figure 3:**
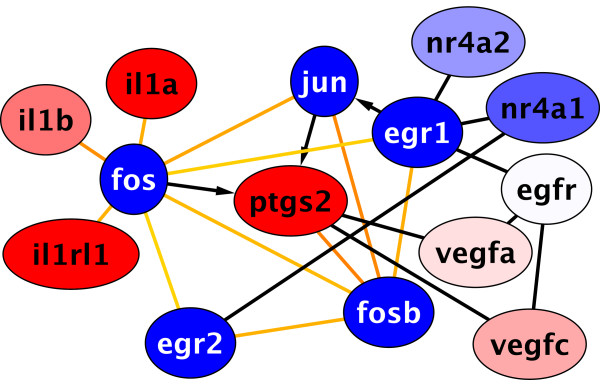
**Pathway analysis of major gene products affected by virulent infection**. A sub-network of the response network from Figure 2 reveals interactions between the major gene products affected by virulent infections. This network is in accordance with Figure 5 of Djavani *et al*. (2007) [16]. In contrast to Djavani *et al*., PTGS2 and IL1RL1 (not shown: as well as IL1R1 and IL1R2) are up-regulated. EGR1, EGR2, FOS, FOSB are transcription factors. PTGS2 encodes prostaglandin-endoperoxide synthase 2, IL1A/B code for interleukin 1 and IL1RL1 belongs to the interleukin 1 receptor family. Color coding is according to Figure 1. Black arrows indicate known connections not realized in this particular response network (see Additional files 5 &6).

With respect to virus response, studies by Djavani *et al*. (2007) report down-regulation of EGR1 and EGR2 after infection with the lymphocytic choriomeningitis virus (LCMV) in a monkey model [16]. Down-regulation of these genes has been recorded by Djavani *et al*. after day 1 until the end of their measurements at day 7. This evidence is consistent with our findings. Thus it seems that virus infection triggers different reactions in the same first responder genes compared to wound healing. Interested to report with this respect is a regulatory network identified by Djavani *et al*. that includes EGR1, EGR2, FOS, JUN and PTGS2 (Figure 3, Additional files 5 &6). In contrast to Djavani *et al*., PTGS2 and IL1RL1 are up-regulated (Not shown: IL1R1 and IL1R2 are also up-regulated). These results indicate moderately different host responses during different viral infections even for such a compact and highly connected network of major host responding genes.

A study of cellular response in birds to H5N1 [17] reports several genes to be significantly regulated. None of the genes reported match the genes found in this study, though several are similar. Many genes, such as SOCS3, IL17C and STAT1 in this study, are similar to the genes SOCS1, IL17A and STAT3 reported in [17]. These genes show opposite regulation in this study compared to the results in birds. Other genes, such as TLR2 in this study and TLR15 in birds are both found to be activated by exposure to H5N1.

Of the strongly regulated genes, several are also found in the SVM analysis described in the last portion of the Methods section. The genes common to the significance analysis and the top 35 components of the SVM analysis are shown in Table 4. The two largest components of the vector describing the optimal hyperplane are for the genes CYR61 and SOCS3. These genes have been associated with ventilator-induced lung injury in mice, along with CCL2 [18]. It has also been found that androgen receptors enhance STAT3, which in turn regulates transcription of SOCS3 [19]. This system is responsible for leptin regulation and may indicate a change in the energy use of a cell when responding to a pathogen. Variations in levels of CYR61 have also been reported in human tumor cell lines from nervous tissue along with a structurally related gene, CTGF [20].

Over-expression of ZFP36 has been found after wounding keratinocytes in human tissue [21], though this is in contrast to the down regulation observed in this study. The study by [21] also shows FOS and EGR1 to be activated by injury, again with opposite results of the data presented here where both are found to be down regulated. Up regulation of ZFP36 has also been reported between bouts of muscular exercise [22]. The reason for the down-regulation of ZPF36 upon exposure to H5N1 while it is up regulated in response to physical injury is probably due to modulation effects caused by the virus infection.

SVM results don't share any of the genes found by BiNGO analysis. That is, no gene was found to be both associated with a significantly over represented GO node and among the most relevant genes in the SVM analysis. Rather, the genes found by SVM tend to be functionally related to many of the genes found in significant GO clusters. These genes include JUN, FOS, FOSB, EGR1, DUSP1, and CYR61. This distinction is likely due to SVM selecting based on differential expression and without regard to relative statistical significance or gene relationships. Curiously, the CCL2 gene that features as a prominent hub in the human network graph is not found to be significant in either the BiNGO or the SVM analysis.

**Figure 4:**
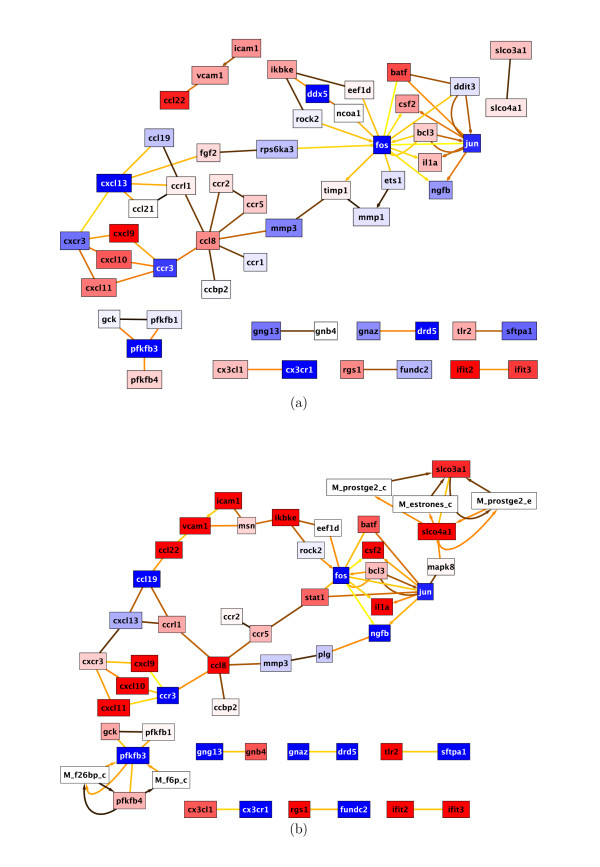
**Differential expression network between H5N1 and RSV**. Response network of differentially expressed genes between H5N1 and RSV. (a) A response network H5N1 versus RSV at 8 h is shown with 54 nodes and 65 interactions. The network was calculated with parameters *k *= 3 and *l *= 4.5. (b) A H5N1 versus RSV response network at 24 h is depicted with 53 nodes and 71 interactions. Parameters *k *= 3 and *l *= 5 were used for calculation. Color coding is according to Figure 1. Edges with arrows indicate chemical reactions, diamond-shaped edge tips denote activation, circle-shaped tips refer to phorphorylation reactions and non-decorated edges are non-directional interactions by physical binding or by inferrence from the iHOP database (see Additional files 7, 8, 9 and 10).

The synthesized networks enable us to determine the regulations (the genes located in up- and down-stream of the pathways) of the selected genes. For example, SAA (serum amyloid protein) 2 and 4 are important host markers for H5N1 HPAI infection. CXCL9, CXCL 10 and 11, CXCR4 along with IL1A regulates expression of SAA proteins that induce immune response molecules specific for H5N1 infection (OAS1, OAS2, OAS3, OASL, GBP1, GBP2, and TAP1), but not RSV or mock infection. Figure 4 shows response networks at 8 h and 24 h after infection between differentially expressed genes of H5N1 and RSV (see Additional files 7, 8, 9 and 10). Already at 8 h, CXCL9 is 3.8 times more expressed in H5N1 than in RSV. This ratio increases to 18.5 at 24 h. Similar increases can be identified for CXCL10 and CXCL11. The relative expression between H5N1 and RSV for these genes change from 2.3 and 2.0 at 8 h to 6.8 and 10.5 at 24 h, respectively. With respect to OAS genes, relative expression of OASL changes from 2.9 to 6.1 between 8 h and 24 h. Interesting to note at 24 h is the ICAM1 triggered two-pronged expressed cascade to the FOS/JUN pair as well as to the chemokine ligand family (CCL, CXCL) and their corresponding receptors (CCR, CXCR). Thus, H5N1 seems to trigger a variety of cytokine response in contrast to RSV infections. The exceptions are CCR3, CXCL13 and CCL19. These chemokines are suppressed by H5N1, indicating a weakened anti-viral response induced by H5N1 infections. The immune responses are further developed into viral specific immune responses by expressing molecules in interferon family. Targeting any of these genes aligned in immune response pathways can boost human anti-viral immune system.

## Conclusions

The expression of approximately 100 genes in human epithelial lung cells is found to be significantly different at 8 and 24 hours after H5N1 exposure compared to normal cellular metabolism or to response to RSV infection. Only those genes found to be significant at 24 hours are significantly clustered in the Gene Ontology with Cell Cycle and various Immune Response nodes being over represented. Although many genes are found to be differentially expressed after exposure to RSV that are not found in either the control or in the H5N1 experiment, no significant clustering was found using the BiNGO analysis. The faster time scales and more intense immunological response may be factors in the virulence of H5N1. These results are consistent with reports from infections caused by other aggressive viruses, such as LCMV. Network responses of major gene products affected by virulent infection seems to be conserved between H5N1 and LCMV infections. Although detailed responses clearly distinguish between different viruses. H5N1 causes host immune response by inducing large number of chemokines at the early stages (24 hour) of infection. However, CXCL13 and CXCL14 that are the keys to develop host anti-viral responses are suppressed. Balance among CXCL chemokines may be important to reduce the H5N1 mediated complications (Figure 4). Future studies looking at time scales from 12-48 hours would be helpful in addition to real-time PCR of the most significant genes found here to better determine their roles in the response to H5N1.

Potential applications of these findings include; (1) the identification early host biomarkers that can be used to detect specifically H5N1 infection during the early stages when the clinical symptoms have not been developed, (2) the selection of host targets to develop therapeutic for H5N1 infection.

## Methods

### Experiments

The gene expression data of epithelial cells were measured by Miltenyi Biotec using an Agilent DNA chip. We used Agilent 60-mer Whole Human Genome Oligo Microarray (with array numbers 251485014481 and 251485014482 resp.), containing approximately 44 K genes and genes candidates. Raw expression files can be accessed at: http://www4.utsouthwestern.edu/ForstLab. NHBE cells were purchased from Lonza (Allendale, NJ. USA), cultured and differentiated as described previously [23]. Briefly, the cells are propagated by plating 2-3 × 10^6 ^cells in a 100 mm collagen coated dish in BEGM media (Lonza, Allendale, NJ. USA) until reaching 70-90% confluency, generally in 3-5 days. NHBEs are detached by low concentration trypsin digestion (Sigma, St. Louis, MO, USA) and plated onto the apical surface of Corning permeable inserts at a cell density of 5 × 10^5 ^in 24 mm (6-well) and 2.5 × 10^5 ^in 12 mm (12-well) in ALI media (50:50 mix of BEGM media and DMEM-H (Sigma, St. Louis, MO, USA) without antibiotics) in both the apical and basolateral chambers. Retinoic acid final concentration in ALI media is at 50 nM. At confluency, the apical media is removed and the cells exposed to air for 28 d. Basolateral media is changed and apical surface washed with 1× PBS every 48 hr. Differentiation is determined by microscopic observation of ciliary beat and confirmed in one-well by formalin-fixed, paraffin embedded hematoxylin and eosin staining for apical ciliary axonemes under microscopic examination.

The fully differentiated cells were infected with High Pathogenic Avian Influenza A (HPAI) H5N1 A/Hong Kong/483/97 in BSL-3 facility with Multiplicity Of Infection (MOI) of 0.01. The infectivity of the virus was determined with a plaque assay prior to the experiments. Apical compartment were washed with warm PBS and the viruses were added to the growth media. After one-hour incubation at 35°C, unattached viruses were removed from the apical compartment and one ml of fresh media was added. Eight and 24 hour after infection the cells were harvested by adding RLT lysis buffer (Qiagen RNeasy Kit) and total RNA was isolated according to the Manufacturer's instructions. One *μ*g of total RNA was used for microarray analysis. The time-points were chosen to record physiological changes during infections. After 8 h the first viral particle start forming inside of host cells. By 24 hours the newly formed viral particles infect the un-infected neighboring cells.

Differential expression levels of mRNA were measured at 8 and 24 hours after exposure to one of three agents. One agent was the H5N1 virus. The other two, which served as controls, were the more benign Respiratory Syncitial Virus (RSV) and Mock samples consisting of growth medium. Three independent experiments were conducted to measure mRNA levels after exposure to H5N1 at both time points, for a total of 6 H5N1 data sets. Two experiments each were carried out for both controls and at both times, for a total of 8 control data sets.

Vials containing human RNA samples were shipped to Miltenyi Biotech on dry ice. At the company, the samples were further quality checked via Agilent 2100 Bioanalyzer platform, T7-base amplified, Cy3/Cy5-labeled and hybridized to the Agilent Whole Human Genome Oligo Microarrays using Agilent's recommended hybridization chamber and oven. Fluorescence signals of the hybridized Agilent Oligo Microarrays were detected using Agilent's DNA microarray scanner. The Agilent Feature Extraction Software (FES) was used to read out and process the microarray image files. Feature intensities and ratios, including background subtraction and normalization, were determined, outliers rejected and statistical confidences (p-values) calculated (see Additional file 11 for microarray statistics).

### Expression Data Analysis

Genes in each of the data sets were deemed as significant if they showed at least a two-fold change in expression and met a preset p-value cutoff. How this value is selected is discussed below. Those genes that were significant in all three exposures to H5N1 after 8 or 24 hours but were not significant in any of the control trials were taken as relevant to H5N1 response. Stated formulaically,(1)

where *E *is the set of genes expressed after H5N1 exposure, *C *is the set of control genes and *S *is the set of genes determined to be significantly relevant to H5N1 response uniquely. The subscripts indicate the time point. Thus, the significantly expressed genes relevant to H5N1 response at a given time point of 8 or 24 hours are taken to be the intersection of all genes significant in all three trials, but not significantly expressed in any of the control trials. In these experiments, the number of probe sets differed between the RSV, H5N1 and Mock conditions. In order to avoid false results due to a probe appearing in one condition but not another, only the probes that were common to all three conditions were kept to be processed by the above analysis.

The timescale of response to RSV and H5N1 may differ substantially, thus no attempt is made to distinguish between controls at 8 hours and those at 24 hours. Any genes that are significant in any of the control studies are assumed to be part of normal cellular metabolism and unrelated to a response to H5N1. This is an oversimplification and will probably lead to several genes in the RSV control studies being incorrectly interpreted as being part of the normal cellular metabolism. What remains, however, can then be more reliably identified as being both significant and genuinely a response of the cell to H5N1. This bias should generally select for genes responding to the more virulent nature of H5N1 since the basic response of the cells to the more benign RSV will be interpreted as normal expression through the control.

To find which genes were significantly expressed as a part of the cellular immune response a conservative interpretation of the data is used. Only genes which are found to be significant in all three experimental trials are considered to be genuine. Thus, a gene must be both biologically and statistically significant in all three experiments at 8 or 24 hours post-exposure to be considered relevant. As mentioned above, the control studies are also given a conservative interpretation. If a gene is found to be both biologically and statistically significant in any one of the control studies then it is interpreted as being part of the normal functioning of the cell. This rather conservative interpretation has the effect of revealing only the most significant genes, and hopefully, the genes that are most important in the reactions of the cell that are most specific to H5N1.

### Evaluation of Significance

A gene is considered biologically significant if there is at least a 2-fold change in the level of gene expression at the given time frame after exposure in all three repeats of the experiment. Statistical significance is determined by demanding that there be, on average, less than one false positive due to statistical effects in the set of genes deemed to be significant. To do this, only data with p-values below a certain cutoff value are deemed to be statistically significant. This cutoff value is(4)

where *N *≈ 3 × 10^4 ^is the number of genes measured. Since the p-value indicates the fraction of random, i.e., insignificant, results that will by chance appear significant, the number of false positives, *FP*, is assumed to be *FP *= *p*_cutoff_(*N *- *TP*), where *TP *is the number of true positives. Since, in general, *TP *≪ *N *it can be approximated that the number of false positives is *FP *≈* p*_cutoff_*N*. By setting a cutoff such that *FP *= 0.5 it can be assumed that, on average, there are less than 0.5 false positives per experiment, i.e., a family-wise error rate of 0.5. Since there are three H5N1 experiments at each time point, and it is required that a result appear significant in all three data sets, the likelihood of a false positive goes down by the cube of the single experiment rate. This is equivalent to a Bonferroni correction, modified for the case where *n *independent measurements are made for each variable. Thus, to maintain a *FP *rate of 0.5 the cutoff given in Equation 4 is used. The tacit assumption here is that *TP *genes will reliably reappear as significant across experiments while *FP *results will be evenly distributed amongst the insignificant genes randomly. This assumption will become important presently.

In the data sets several genes are listed twice or more. These repeats are due to different oligonucleotide probe sequences being used on the gene chip that code for different parts of the same gene. Genes that appeared more that once were not all necessarily measured to be (in)significant in all instances. In the cases where the results on a single chip differed, a way of evaluating the significance of that gene is needed. In these cases, if a gene appeared as significant by p-value more than once, and its average fold change over all instances was greater than a factor of two, then it was taken to be significant.

Since genes which are repeated have more chances to appear significant the cutoff p-value given above may not be sufficient. If a gene appears beneath the p-value cutoff twice, then the FP rate will be less than *p*^2^. As the number of instances of the gene that are not measured to be significant increases, however, there is a greater chance the gene is not significant. To determine the proper outcome the false negative rate, *FN*, must be known. This quantity is unknown but is tacitly assumed by the above procedure to be *FN *≫ , making two positive occurrences compelling. The precise *FN *rate is complicated since the redundant entries represent diffferent probe sequences for the same gene. The average fold change of all instances is used in determining biological significance to avoid biasing the measure. This is not a rigorous statistical treatment, but should provide reasonable results in conjunction with the other data sets and for reasonable values of a *FN *rate.

Changing the p-value cutoff described above did not appreciably change the numbers of significant genes identified. With respect to the H5N1 response after 8 h and 24 h, a total of 152 and 209 genes, respectively, satisfy Equation 3 when the p-value cut-off is set to unity. These numbers change to 138 and 213 genes when, in addition, the p-value cutoff in Equation 4 is used. The increase from 209 to 213 genes at 24 hours after the inclusion of the p-value cutoff reflects the cutoff eliminating genes from being considered significant in the control study. All but about 20 genes in both time sets remained significant regardless of what p-value cutoff was used in the range *p*_cutoff _<*p *< 1.0.

### Gene Ontology

The genes found to be significant in the experiments involving H5N1 exposure are analyzed using the BiNGO package for CytoScape. Here, a list of significantly over-represented Gene Ontology nodes associated with the significant genes is constructed. Similar to enrichment analysis [24], the Gene Ontology is then searched for nodes that are over represented at a statistically significant level. Significance is evaluated using the hypergeometric test given by

where *q*_*i *_gives the probability for a given GO node having *i *of its *f *daughter nodes associated with genes from a random list of *c *genes, and *g *is the number of GO nodes in the whole Ontology. Thus, the chance of *n *or more genes randomly being clustered under a given GO node (given that *c *genes were found to be significant) has a probability of(5)

Otherwise stated, this is the probability of finding a clustering at least as extreme by random chance, i.e. the p-value of the clustering.

### Human Network Reconstruction

To construct a hybrid *Homo sapiens *interaction and reaction network we have combined protein-protein interactions with directional signal transduction and metabolic reactions. We have been using interaction information from IntAct [25], NetworKin [26] and from Palsson's group, consisting of 37,000 nodes (genes, proteins and small chemicals) as well as 156,000 interactions (gene-protein, protein-protein) and reactions (chemical, protein-phosphorylation, etc). We have also integrated this network with a larger literature based network available from iHOP [27] with 45,041 nodes and 438,567 interactions, which are already about 2/3s of 650,000 interactions predicted by Stumpf *et al*. (2008) [28]. As a third reference network, the Homo sapiens protein interaction network was downloaded from the BioGRID database version 2.0.39 [29], which was generated from literature curation of protein interaction data. The data set was filtered to include only direct and physical interactions between human proteins and all loops and duplicate edges were removed. Although, duplicate edges from different data sources and different property (e.g., an interaction identified as generic protein-protein interaction in one data-set and predicted as phosphorylation of a protein by a kinase in another data-set) were kept to emphasize the importance/validity of such interactions.

### Human Response Network Analysis

The list of genes *S*_8 _and *S*_24 _are used as seed nodes to create so called response networks using the Human Network. The *k*-shortest paths between all pairs of seed nodes are found, where *k *is a positive integer. The length of a path is weighted at each edge by the average absolute log-fold change in expression of the two genes at either end. The parameter *k *is chosen such that the graphs showed a large number of interaction pathways while remaining easily interpretable. The reader is referred to Cabusora *et al*. (2005) [30] where the algorithm is described in detail.

Within the Human Network, 37 of the 8 hour nodes and 80 nodes in the 24 hour set were identified. The degree to which each gene is interconnected and with which genes they interact can help identify what role they play in the cell's response to H5N1. These networks can also help identify important centers of activity such as the FOS and EGR1 genes seen in Figure 1.

In the Human Networks generated using the genes found in the BiNGO clustering analysis as seeds it is desirable to know how often particular genes are added when Human Networks are constructed. The probability a given gene will be added to a human network given a random list of seed genes is determined using Monte Carlo simulations for calculating the p-values of the appearance of genes. The p-values calculated are not significantly different for the top several genes for 1000 or 10000 iterations. In general, the distribution is consistent within the first 1000 and afterward statistical noise is reduced allowing better accuracy and resolution of smaller values. Genes with no appearances in the Monte Carlo can only be said to have an estimated p-value of less than 1/interactions. The number of seed genes used in the Monte Carlo simulations was 213, the number found in the significance analysis. The most significant GO nodes and their associated raw p-values are given in Table 6. The p-values for the genes included by the Human Network, not including the seed genes, are listed in Table 7.

**Table 6:** Most significant GO nodes

Significant GO Nodes at 24 Hours												
**GO-ID**		**p-value**		**corr p-val**		**sel**.		**tot**.		**Description**		**Genes in test set**

2376		2.4009E-16		1.8007E-13		35		783		immune system		IFIH1, IFITM1, IFITM3, G1P3, OAS3, PRKR
										process		TLR2, ISG15, OAS1, C1S, OAS2, ISGF3G
												CXCL11, CCL5, HSH2D, BF, PGLYRP4 TAP1
												MX1, SPON2, MX2, EDG6, EXO1, EGR1, ZFP36
												CHST4, HLA-DQA2, AIM2, PDCD1LG1, IKBKE, CD34
												IRF7, IFIT5, DMBT1, GBP1
6955		6.2296E-16		4.6722E-13		31		613		immune		IFIH1, IFITM1, IFITM3, TLR2, G1P3, OAS3
										response		PRKR, ISG15, OAS1, C1S, OAS2, ISGF3G
												CXCL11, CCL5, BF, PGLYRP4, TAP1, MX1
												SPON2, MX2, EDG6, EXO1, CHST4, HLA-DQA2, AIM2
												PDCD1LG1, IKBKE, IRF7, IFIT5, DMBT1, GBP1
51869		4.4363E-13		3.3273E-10		54		2335		response to		PIR51, PRKR, G1P3, TLR2, ISG15, ISGF3G
										stimulus		CXCL11, CALB1, FOS, PGLYRP4, MX1, SPON2
												FANCA, CCNA2, MX2, IHPK3, CYR61, RAMP
												EDG6, ZFP36, ECGF1, SAA4, CHST4, FOSB
												HLA-DQA2, RAD51, PDCD1LG1, UHRF1, GADD45B
												PROS1, GBP1, IFIH1, IFITM1, IFITM3
												OAS3, OAS1, CHEK1, OAS2, C1S, CCL5
												BF, TYMS, TAP1, EXO1, GSTA3, SGK, STAT1
												ABCG1, AIM2, IKBKE, DUSP1, IRF7, IFIT5, DMBT1
7049		1.8839E-10		1.4129E-7		28		808		cell cycle		E2F1, PRC1, IFITM1, HCAP-G, PRKR, CHEK1
												MCM10, CCNE2, SPC25, CDC45L, CCNA2
												EXO1, CDC6, MKI67, NUSAP1, FOSB, STAT1
												MCM6, RAD51, UHRF1, PLK3, DUSP1 KNTC2
												JUN, TOPK, FOXC1, GADD45B, DMBT1
22402		4.4621E-9		3.3466E-6		24		690		cell cycle		EXO1, E2F1, CDC6, IFITM1, MKI67, PRC1
										process		HCAP-G, NUSAP1, CHEK1, FOSB, MCM10, STAT1
												RAD51, CCNE2, SPC25, PLK3, CDC45L, KNTC2
												JUN, TOPK, FOXC1, GADD45B, CCNA2, DMBT1
9615		5.9766E-9		4.4825E-6		10		91		response		IFIH1, IRF7, PRKR, ISG15, OAS1, ISGF3G
										to virus		CCL5, MX1, STAT1, MX2
6260		8.9240E-9		6.6930E-6		13		188		DNA		EXO1, CDC6, ECGF1, MCM10, ORC1L, TK1
										replication		RAD51, MCM6, CCNE2, TYMS, CDC45L, PFS2, RAMP
9607		2.1548E-8		1.6161E-5		14		241		response to		IFIH1, IFITM1, IFITM3, PRKR, ISG15, OAS1
										biotic stimulus		ISGF3G, STAT1, CCL5, PGLYRP4, IRF7, MX1
												MX2, DMBT1
51707		5.8349E-8		4.3762E-5		12		182		response to		IFIH1, PGLYRP4, IRF7, PRKR, ISG15, OAS1
										other organism		ISGF3G, CCL5, MX1, STAT1, MX2, DMBT1
6950		5.9053E-7		4.4290E-4		24		894		response to		EXO1, ZFP36, GSTA3, PIR51, SGK, TLR2
										stress		SAA4, CHST4, CHEK1, C1S, CCL5, CXCL11, RAD51
												BF, TYMS, FOS, UHRF1, DUSP1, GADD45B, PROS1
												CCNA2, FANCA, IHPK3, RAMP
74		1.7011E-6		1.2758E-3		17		506		reg. of prog.		E2F1, CDC6, MKI67, IFITM1, NUSAP1, CHEK1
										through cell cycle		FOSB, STAT1, MCM10, CCNE2, PLK3, CDC45L
												JUN, FOXC1, GADD45B, CCNA2, DMBT1
51726		1.8935E-6		1.4202E-3		17		510		regulation of .		E2F1, CDC6, MKI67, IFITM1, NUSAP1, CHEK1
										cell cyc		FOSB, STAT1, MCM10, CCNE2, PLK3, CDC45L
												JUN, FOXC1, GADD45B, CCNA2, DMBT1
6270		3.1656E-6		2.3742E-3		5		27		DNA replication initiation		CCNE2, CDC6, CDC45L, ORC1L, MCM6
51706		6.8716E-6		5.1537E-3		12		285		multi-organism		IFIH1, PGLYRP4, IRF7, PRKR, ISG15, OAS1
										process		ISGF3G, CCL5, MX1, STAT1, MX2, DMBT1
6259		9.7883E-6		7.3412E-3		19		704		DNA metabolic		EXO1, CDC6, PIR51, ECGF1, CHEK1, MCM10
										process		ORC1L, MCM6, TK1, RAD51, CCNE2, TYMS, FOS
												UHRF1, CDC45L, PFS2, FANCA, IHPK3, RAMP
6263		1.9790E-5		1.4843E-2		7		96		DNA-dependent DNA replic.		EXO1, CCNE2, CDC6, CDC45L, ORC1L, MCM6, RAD51
278		2.0740E-5		1.5555E-2		11		267		mitotic cell cycle		E2F1, SPC25, CDC6, KNTC2, PRC1, HCAP-G
												NUSAP1, TOPK, FOXC1, CHEK1, CCNA2
9719		2.9713E-5		2.2285E-2		12		330		response to		EXO1, TYMS, UHRF1, PIR51, SGK, CHEK1
										endogenous stim.		FANCA, CCNA2, IHPK3, ABCG1, RAD51, RAMP
6952		4.3825E-5		3.2869E-2		15		520		defense response		ZFP36, IFIH1, TLR2, SAA4, CHST4, C1S
												CXCL11, CCL5, BF, FOS, PGLYRP4, TAP1, MX1
												MX2, DMBT1
6974		4.8613E-5		3.6459E-2		11		293		response to DNA		EXO1, TYMS, UHRF1, PIR51, SGK, CHEK1
										damage stim.		FANCA, CCNA2, IHPK3, RAD51, RAMP
279		5.9994E-5		4.4996E-2		10		248		M phase		EXO1, SPC25, CDC6, KNTC2, HCAP-G, NUSAP1
												TOPK, CHEK1, CCNA2, RAD51
22403		6.1999E-5		4.6500E-2		11		301		cell cycle phase		EXO1, E2F1, SPC25, CDC6, KNTC2, HCAP-G
												NUSAP1, TOPK, CHEK1, CCNA2, RAD51

The most significant GO nodes are given with their raw p-values. Under each GO entry is a list of the genes associated with that node.

**Table 7:** Genes found in the Human iHOP Network using genes significant at 24 hours as seeds

Gene	p-value
CCL2	4.0 × 10^-1^
CSF2	1.0 × 10^-2^
CSF3	3.8 × 10^-2^
CXCL10	1.4 × 10^-1^
CXCR4	8.1 × 10^-3^
IL1a	3.2 × 10^-1^
PTGS2	2.1 × 10^-2^
SOD2	1.0 × 10^-1^
TLR1	< 1.0 × 10^-4^
TNF	1.7 × 10^-2^

The network generated from the BiNGO results show several closely related genes to be networked with the genes found in the significance analysis. Interestingly, there is no correlation between the connectivity of a gene in the Human Network and the p-value of that gene appearing in the human network given a random set of seed genes. Neither the first nor the second order connectivity of the nodes is correlated with the gene p-values. How important the higher order connectivities are, however, depends on the maximum path-length allowable. Second, the weights in the expression data will alter the weight of including each gene in a path. Thus, genes that are only moderately well connected but have a low cost of inclusion may appear more frequently than genes that are better connected but with a higher cost of inclusion.

### Support Vector Machines

To provide a check against the significance analysis described above, the data are also examined using Support Vector Machines (SVM). SVM is a technique used in machine learning that is designed to classify objects into one of two classes [31]. Each object has a number of parameters describing it that have been measured. In the case presented here the set of measured parameters is the set of gene expression values in one experiment, i.e., where each gene's level of expression constitutes one parameter. Each object, i.e., experiment, can be plotted in Cartesian coordinates of a dimension equal to the number of parameters, with each axis encoding the value of one parameter. I this implementation the gene list is limited to those genes with a p-value of 0.05, or about twice *p*_cutoff _or less. This helps avoid fitting to noise. Using this cutoff results in having 14 points plotted in a space of 332 dimensions rather than the total 3 × 10^4^. Thus, each experiment can be represented in this space as a vector whose components describe the degree of regulation for each gene measured. Once all the objects are plotted, it is often possible to draw a hyperplane that divides the space so that all objects of one class are found one one side of the hyperplane and all objects of the other class are on the other side. Such a hyperplane could then be used to classify the results from a gene chip and help determine whether the results indicated the cell had been exposed to H5N1.

In this case the components of the hyperplane itself are examined rather than using it to classify unknown objects. The vector normal to the Optimal Hyper Plane (OHP) separating the two classes of objects is used to determine which genes may be important in cellular response to H5N1. The hyperplane separating the two groups (control and H5N1 exposures) maximizes the margin between the groups, i.e., the distance perpendicular to the OHP separating the nearest members of the two groups. Thus, the largest components of this vector are the directions in which the margin between the control and exposed groups is greatest. A list of the genes whose change in regulation show the greatest margin between the two groups is then compiled. The list of genes used in the SVM analysis is restricted to those with p-values of 0.05 or less. This is to avoid fitting as much statistical noise as possible. In spite of no cutoff for the fold change being required explicitly, the most prominent genes in the SVM analysis have fold changes that are generally more than a factor of two and p-values that are typically much less than the cutoff. The results are shown in Table 5 and can be compared to the results from the analysis described in Evaluation of Significance. Based on the average p-values of the genes listed, the expected number of false positives among the 35 genes listed is only around 0.3.

## Authors' contributions

KT carried out the computational biology studies and performed the statistical and network analysis as well as drafted the manuscript. AZ carried out the BSL-2 molecular biology experiments and performed initial gene expression analysis. RH carried out the iHOP interaction prediction and provided the iHOP network. KSH performed the BSL-3 infection experiments and mRNA isolation for expression profiling. KSH, RMR and CVF conceived of the study and participated in its design. CVF further performed additional respone network analysis, helped to draft and finalized the manuscript. All authors have read and have approved the final manuscript.

## Supplementary Material

Additional file 1 **Figure **1 **network data**. Figure 1 network data in XML format Click here for file

Additional file 2 **Figure **1** interaction data**. Figure 1 interaction data in sif format Click here for file

Additional file 3 **Figure **2** network data**. Figure 1 network data in XML format Click here for file

Additional file 4 **Figure **2** interaction data**. Figure 2 interaction data in sif format Click here for file

Additional file 5 **Figure **3 **network data**. Figure 3 network data in XML format Click here for file

Additional file 6 **Figure **3 **interaction data**. Figure 3 interaction data in sif format Click here for file

Additional file 7 **Figure **4a** network data**. Figure 4a network data in XML format Click here for file

Additional file 8 **Figure **4a** interaction data**. Figure 4a interaction data in sif format Click here for file

Additional file 9 **Figure **4b **network data**. Figure 4b interaction data in XML format Click here for file

Additional file 10 **Figure **4b** interaction data**. Figure 4b interaction data in sif format Click here for file

Additional file 11 **Supplemental Data**. Statistics of microarray experiments Click here for file
