# Supplementary material for: Response network analysis of differential gene expression in human epithelial lung cells during avian influenza infections
Source: BMC Bioinformatics. 2010 Apr 6;11:170. doi: 10.1186/1471-2105-11-170 (PMC2868837; doi:10.1186/1471-2105-11-170)
Supplement: Additional file 11 — Supplemental Data. Statistics of microarray experiments [file 1471-2105-11-170-S11.PDF]

Supplemental Data

Response network analysis of differential gene expression in human epithelial lung cells during avian influenza infections

Ken Tatebe, Ahmet Zeytun, Ruy M. Ribeiro, Robert Hoffman, Kevin S. Harrod, Christian V. Forst

1 RSV and Mock Response

1.1 RSV Response Microarray Statistics

	RSV							
Slides	0h 1	8h 1	0h 1	24h 1	0h 2	8h 2	0h 2	24h 2
Total Number of Features	45015		45015		45015		45015	
Number of Found Features*	41067		40892		40443		40498	
# of Features with Significant Signal†	31150	33165	31635	34148	31664	31379	31931	33463
# of Non-Uniformity Outliers	89	73	71	48	25	52	15	18
# of Population Outliers	264	249	204	176	213	202	246	222
# Saturated Features: SpikeIns	0	0	0	0	0	0	0	0
# Saturated Features: Non-Ctrl	0	2	0	3	0	2	0	0
Absolute Average Log-Ratio: SpikeIns	0.41		0.43		0.44		0.43	
Standard Dev. Log-Ratio: SpikeIns	0.02		0.02		0.02		0.01	
Average S/N: SpikeIns	27.95		26.91		29.27		37.66	
Absolute Average Log-Ratio: Non-Ctrl	0.16		0.15		0.13		0.09	
Standard Dev. Log-Ratio: Non-Ctrl	0.04		0.04		0.03		0.03	
Average S/N: Non-Ctrl	10.25		7.59		8.24		6.45	

*Ratio of (feature signal)/(local BG signal) $> 1.5\times$(local BG noise); and spot$\emptyset \geq 0.03\times$(nominal spot$\emptyset$)
†according to the Agilent estimation of background error model

1.2 Mock Response Microarray Statistics

	Mock							
Slides	0h 1	8h 1	0h 1	24h 1	0h 2	8h 2	0h 2	24h 2
Total Number of Features	45015		45015		45015		45015	
Number of Found Features*	40822		41070		40378		40530	
# of Features with Significant Signal†	30394	30527	31639	32975	31163	32211	31601	33428
# of Non-Uniformity Outliers	11	40	15	30	15	12	26	48
# of Population Outliers	183	161	137	126	117	112	182	216
# Saturated Features: SpikeIns	0	0	0	0	0	0	0	0
# Saturated Features: Non-Ctrl	0	3	0	1	0	2	0	2
Absolute Average Log-Ratio: SpikeIns	0.43		0.44		0.45		0.44	
Standard Dev. Log-Ratio: SpikeIns	0.01		0.01		0.01		0.01	
Average S/N: SpikeIns	44.57		41.48		45.13		37.91	
Absolute Average Log-Ratio: Non-Ctrl	0.03		0.22		0.03		0.08	
Standard Dev. Log-Ratio: Non-Ctrl	0.04		0.03		0.03		0.03	
Average S/N: Non-Ctrl	2.89		16.79		2.45		6.07	

*Ratio of (feature signal)/(local BG signal) $> 1.5\times$(local BG noise); and spot$\emptyset \geq 0.03\times$(nominal spot$\emptyset$)
†according to the Agilent estimation of background error model

1.3 RSV Scatter plots

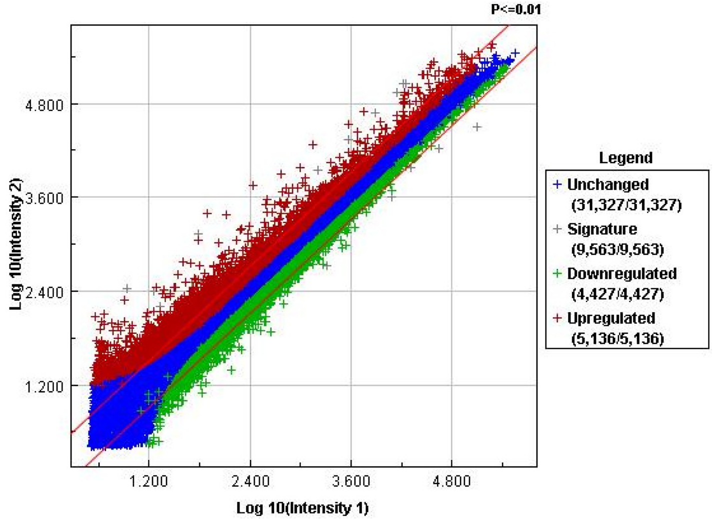

(A)

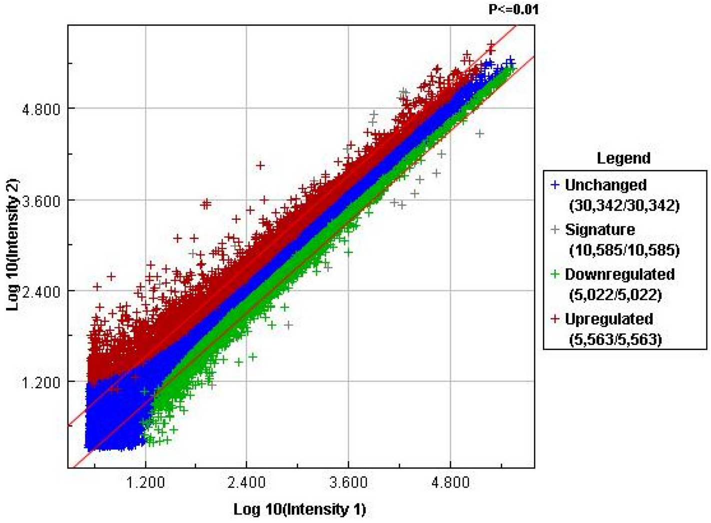

(C)

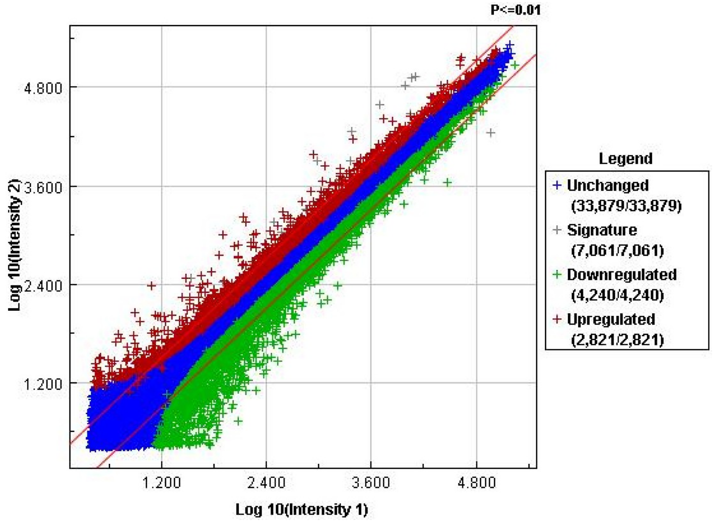

(B)

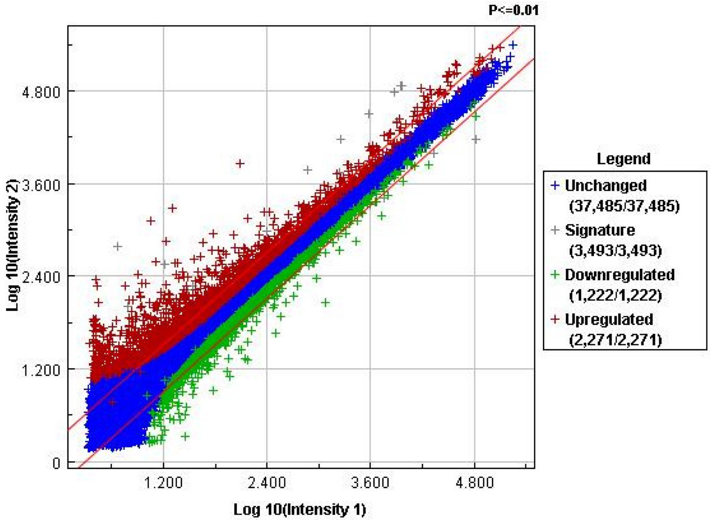

(D)

Figure 1: RSV scatter plots of signal intensities (C.f. Microarray service report by Miltenyi Biotec). The signal intensities of each feature represented by a dot is shown in double logarithmic scale. X-axis: Cy3-log signal intensity; y-axis: Cy5-log signal intensity. Red diagonal lines define the areas of 2-fold differential signal intensities. Blue cross: unchanged genes. Red cross: significantly upregulated genes (p-value < 0.01). Green cross: significantly downregulated genes (p-value < 0.01). Grey cross in legend: summary of significantly up- and downregulated signatures. (A) 0h RSV_1 versus RSV_1 at 8h; (B) 0h RSV_2 versus 8h RSV_2; (C) 0h RSV_1 versus 24h RSV_1; (D) 0h RSV_2 versus 24h RSV_2.

1.4 Mock Scatter plots

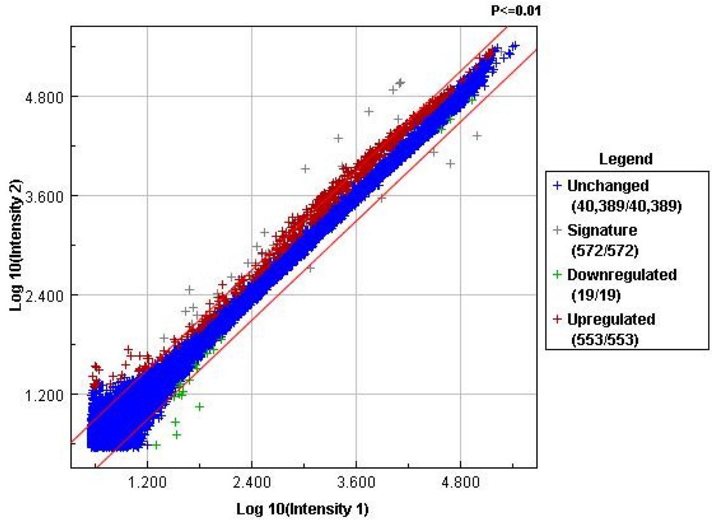

(A)

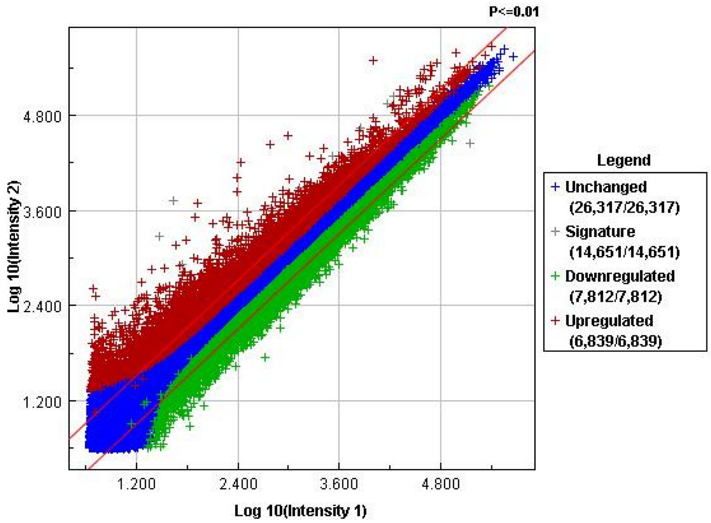

(C)

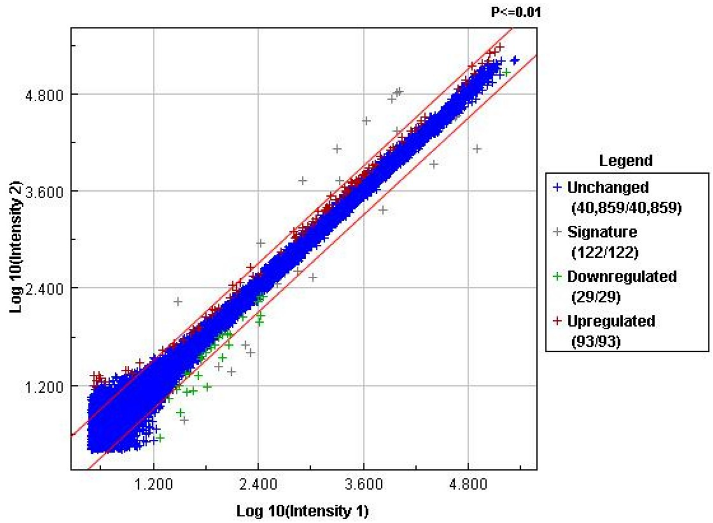

(B)

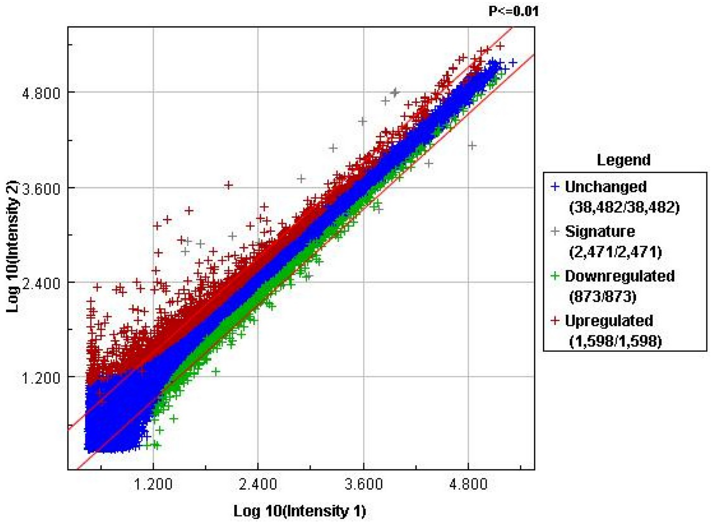

(D)

Figure 2: Mock infection scatter plots of signal intensities (C.f. Microarray service report by Miltenyi Biotec - see Figure caption of Figure 1). (A) 0h Mock_1 versus 8h Mock_1; (B) 0h Mock_2 versus 8h Mock_2; (C) 0h Mock_1 versus 24h Mock_1; (D) 0h Mock_2 versus 24h Mock_2.

2 H5N1 Response

2.1 H5N1 Response Microarray Statistics

	H5N1											
Slides	0h 1	8h 1	0h 2	8h 2	0h 3	8h 3	0h 1	24h 1	0h 2	24h 2	0h 2	24h 2
Total Number of Features	45015		45015		45015		45015		45015		45015	
Number of Found Features*	41840		41582		42301		42891		41392		41616	
# of Features with Significant Signal†	38807	38610	37303	37488	37765	40976	39725	42466	38007	38908	37901	36276
# of Non-Uniformity Outliers	4	35	6	37	13	31	8	20	20	134	61	121
# of Population Outliers	49	56	62	42	70	43	77	55	53	78	30	39
# Saturated Features: SpikeIns	0	0	0	0	0	0	0	0	0	0	0	0
# Saturated Features: Non-Ctrl	0	1	0	1	0	1	0	1	0	1	0	2
Absolute Average Log-Ratio: SpikeIns	0.47		0.50		0.47		0.46		0.47		0.47	
Standard Dev. Log-Ratio: SpikeIns	0.01		0.01		0.01		0.02		0.01		0.01	
Average S/N: SpikeIns	54.49		47.40		37.44		40.77		43.47		45.89	
Absolute Average Log-Ratio: Non-Ctrl	0.11		0.10		0.11		0.12		0.14		0.13	
Standard Dev. Log-Ratio: Non-Ctrl	0.03		0.03		0.03		0.02		0.03		0.03	
Average S/N: Non-Ctrl	9.49		9.17		8.48		10.51		12.55		10.99	

*Ratio of (feature signal)/(local BG signal) $>$ 1.5$\times$(local BG noise); and spot$\emptyset \geq 0.03\times$(nominal spot$\emptyset$)

†according to the Agilent estimation of background error model

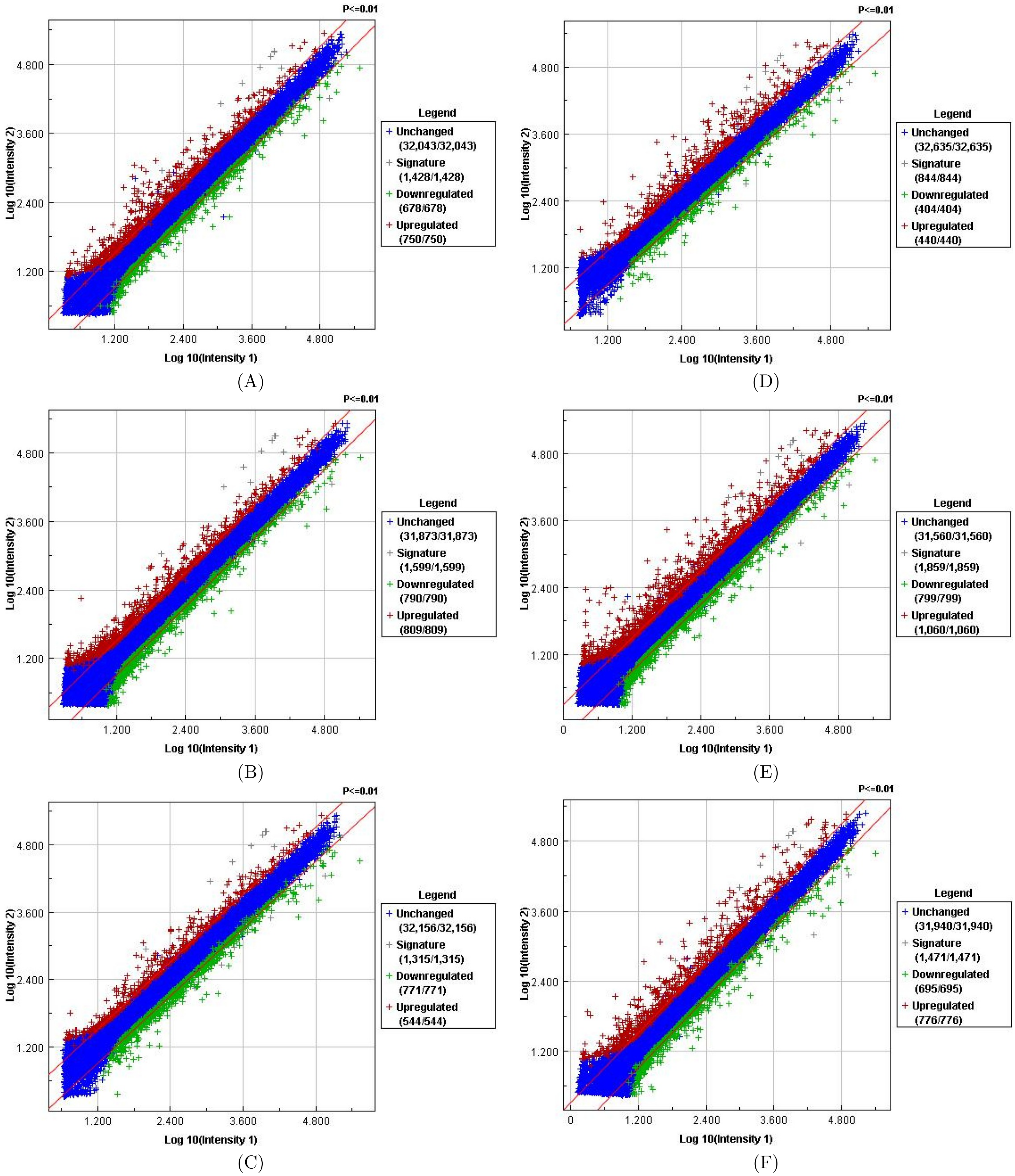

Figure 3: H5N1 scatter plots of signal intensities (C.f. Microarray service report by Miltenyi Biotec - see Figure caption of Figure 1). (A) 0h H5N1$_1$ versus 8h H5N1$_1$; (B) 0h H5N1$_2$ versus 8h H5N1$_2$; (C) 0h H5N1$_3$ versus 8h H5N1$_3$; (D) 0h H5N1$_1$ versus 24h H5N1$_1$; (E) 0h H5N1$_2$ versus 24h H5N1$_2$; (F) 0h H5N1$_1$ versus 24h H5N1$_1$.
